# Atypical Presentation of Metastasis of Appendiceal Cancer: Neoplastic Growth Infiltrating an Inguinal Hernia

**DOI:** 10.1002/ccr3.70870

**Published:** 2025-09-08

**Authors:** Peter Mounas, Tony Elias, Amit Kharod, Nikolette Zamarra

**Affiliations:** ^1^ Rowan‐Virtua School of Osteopathic Medicine Stratford New Jersey USA; ^2^ Department of Surgery CentraState Healthcare System Freehold New Jersey USA

**Keywords:** chronic diseases, gastroenterology/hepatology, oncology, surgery

## Abstract

Although rare, appendiceal cancer can metastasize to unusual sites, including the inguinal canal. In patients with a history of abdominal malignancy presenting with an inguinal hernia, metastatic disease should be considered for adequate recognition and early oncologic intervention.

## Graphical abstract

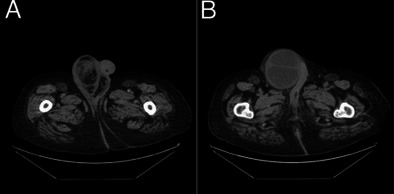

Although uncommon, appendiceal cancer has shown an increasing incidence over the past three decades, necessitating further clinical awareness. The rarity of the diagnosis and its ambiguous presentations can significantly hinder the ability to recognize it in clinical practice. Here, we report a case of primary appendiceal cancer that progressed to pseudomyxoma peritonei with metastasis to the inguinal canal, manifesting as an incarcerated inguinal hernia. Abdominal/pelvic computed tomography (CT) visualized an apparent inguinal hernia of the bowels. Upon surgical excision, neoplastic debris was incidentally discovered. Pathological results were significant for metastatic mucinous carcinoma. Despite tumor resection and hyperthermic intraperitoneal chemotherapy (HIPEC), the risk of unrecognized metastasis remained due to the hernia sac's peritoneal origin and the high tendency of appendiceal cancer to recur in the intraperitoneal cavity. This case underscores the importance of considering metastatic disease in patients with appendiceal malignancy presenting with hernias. It also highlights the potential for delayed diagnosis and further oncologic care in patients who present with typical surgical pathologies such as inguinal hernias.

## 1 Introduction

Appendiceal cancer is a rare malignancy that can pose diagnostic challenges due to its uncharacteristic and nonspecific presentations. Studies have shown that the incidence of appendiceal cancer gradually increased between 2004 and 2017, mostly in patients below the age of 49 [1, 2]. Survival disparities exist, with African American patients experiencing worse outcomes than Hispanic and non‐Hispanic White patients [3]. Appendiceal cancer can begin as a mucocele along the appendix that, with rupture, can cause seeding to the peritoneum and surrounding structures [4]. It is uncertain why appendiceal cancers frequently metastasize, but one report suggests that the thin muscle layer of the appendix may allow significant progression past the serosa layer [5]. One subtype of appendiceal cancer, mucinous adenocarcinoma, is particularly prone to progression to pseudomyxoma peritonei, a condition characterized by localized or generalized accumulation of thick, gelatinous material consistent with neoplasia [6]. Although recurrence rates are known to be high in mucinous adenocarcinoma, they are also significantly high across varying histologies of appendiceal cancer, including low‐grade neoplasms, high‐grade neoplasms, and more severe subtypes such as goblet cell carcinoma, even after successful cytoreductive surgery with hyperthermic intraperitoneal chemotherapy (CRS/HIPEC). Nikiforchin et al. highlight that intraperitoneal recurrence was the most common pattern among the varying subtypes, although high‐grade subtypes had a higher likelihood of extraperitoneal recurrence prior to intraperitoneal recurrence [7]. This broader understanding emphasizes that peritoneal recurrence is not limited to mucinous adenocarcinomas and requires heightened vigilance for metastasis, including patients who had previously received successful HIPEC therapy.

Previous case reports have described appendiceal cancer metastasizing to surrounding structures such as the right colon, stomach, and bladder after invading the peritoneum. For example, a case report from Japan discussed a patient who, despite having undergone adequate surgery for pseudomyxoma peritonei of appendiceal cancer, later presented with anorexia and vomiting due to gastric metastasis that required further resections [8]. Similar reports have shown primary appendiceal cancer with metastasis to the urethra and bladder, causing hematuria and potential blockage of the urinary outflow, resulting in a significantly reduced prognosis [5]. Although the literature on metastasis presenting as a hernia is scarce, one report discusses a patient with manifestations of pseudomyxoma peritonei in an umbilical hernia that was diagnosed by ultrasound‐guided biopsy [9]. These varying presentations underscore the diagnostic challenge of metastatic appendiceal cancer as it can share overlapping clinical features with common surgical diagnoses such as hernias.

Management of pseudomyxoma peritonei usually requires cytoreductive therapy and hyperthermic intraperitoneal chemotherapy (HIPEC), which, in combination, have been shown over the last 30 years to have significant positive therapeutic outcomes in the management of both appendiceal and colorectal tumors [10, 11, 12]. Limited reports have been published concerning the potential of metastasis to the inguinal canal or the potential of presenting as an umbilical or inguinal hernia. A few instances have been reported regarding Amyand's hernia, a condition in which a portion of the appendix is enveloped within the right inguinal hernia sac. Responsible for about 1% of hernia cases, Amyand's hernias can present even more rarely as appendicitis or a tumor within the hernia sac. In such cases, diagnosis is frequently confirmed incidentally through hernia surgery, offering a similar presentation to the one discussed in this report [13, 14, 15, 16].

In this case report, we discuss a distinctive, complex case of a patient with a history of appendiceal cancer who presented with an incarcerated inguinal hernia infiltrated by neoplastic material.

## 2 Case History/Examination

A patient in his 60s presented to the emergency department due to failure to thrive and suprapubic abdominal pain. Four months before admission, the patient had undergone extended right hemicolectomy with primary anastomosis, subtotal gastrectomy with Billroth II reconstruction, splenectomy, liver wedge resection, abdominal debulking, omentectomy, and hyperthermic intra‐abdominal chemotherapy, followed by exploratory laparotomy and extensive lysis of adhesions for pseudomyxoma peritonei secondary to primary appendiceal cancer. Gastrectomy was subsequently completed with the takedown of Billroth II gastrojejunostomy and Roux‐en‐Y esophagojejunostomy with primary repair of colotomy 2 months before admission. The patient had a medical history significant for colon cancer, hypertension, hyperlipidemia, hypothyroidism, and urinary retention. He also had a substantial history of alcohol use but denied smoking or illicit drug use.

On admission, he was tachycardic (110 bpm) with dry mucous membranes. Abdominal examination revealed multiple well‐healed surgical scars, mild diffuse suprapubic tenderness, and a chronic, incarcerated right groin bulge without erythema.

## 3 Methods (Differential Diagnosis, Investigations, and Treatment)

**FIGURE 1:**
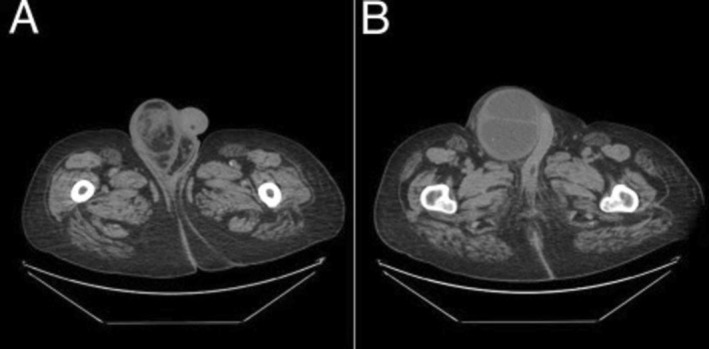
(A, B) Computed tomography of the abdomen and pelvis without contrast showing a large right inguinal hernia.

The diagnosis of an incarcerated inguinal hernia was suspected based on clinical and previous CT findings of the abdomen and pelvis showing a large right inguinal hernia containing a dilated bowel loop, as shown in Figure 1A,B. Other differentials included lymphadenopathy, lymphoma, abscess, hematoma, and metastatic neoplasm.

Upon investigation, pertinent abnormal complete blood count (CBC), complete metabolic panel (CMP), and urinalysis values are shown in Table 1.

**TABLE 1:** Abnormal lab values with corresponding normal reference values for the patient.

Lab	Result	Normal levels
**Complete blood count**		
Red Blood Cells (RBC)	3.4 10 × 6/μL	4.3–5.9 10 × 6/μL
Hemoglobin	10.0 g/dL	Male: 13.5–17.5 g/dL
Hematocrit	30.8%	Male: 41%–53%
RDW	17.1%	11.5%–14.5%
**Complete metabolic panel**		
Albumin	3.3 g/dL	3.5–5.5 g/dL
Alkaline Phosphate	292.0 U/L	25–100 U/L
**Urinalysis**		
Appearance	Cloudy	Clear
Protein	1+	Negative to trace
Nitrates	Positive	Negative
Leukocyte esterase	3+	Negative
Bacteria	1+	Negative

The patient was then admitted for administration of total parenteral nutrition via a peripherally inserted central catheter line.

**FIGURE 2:**
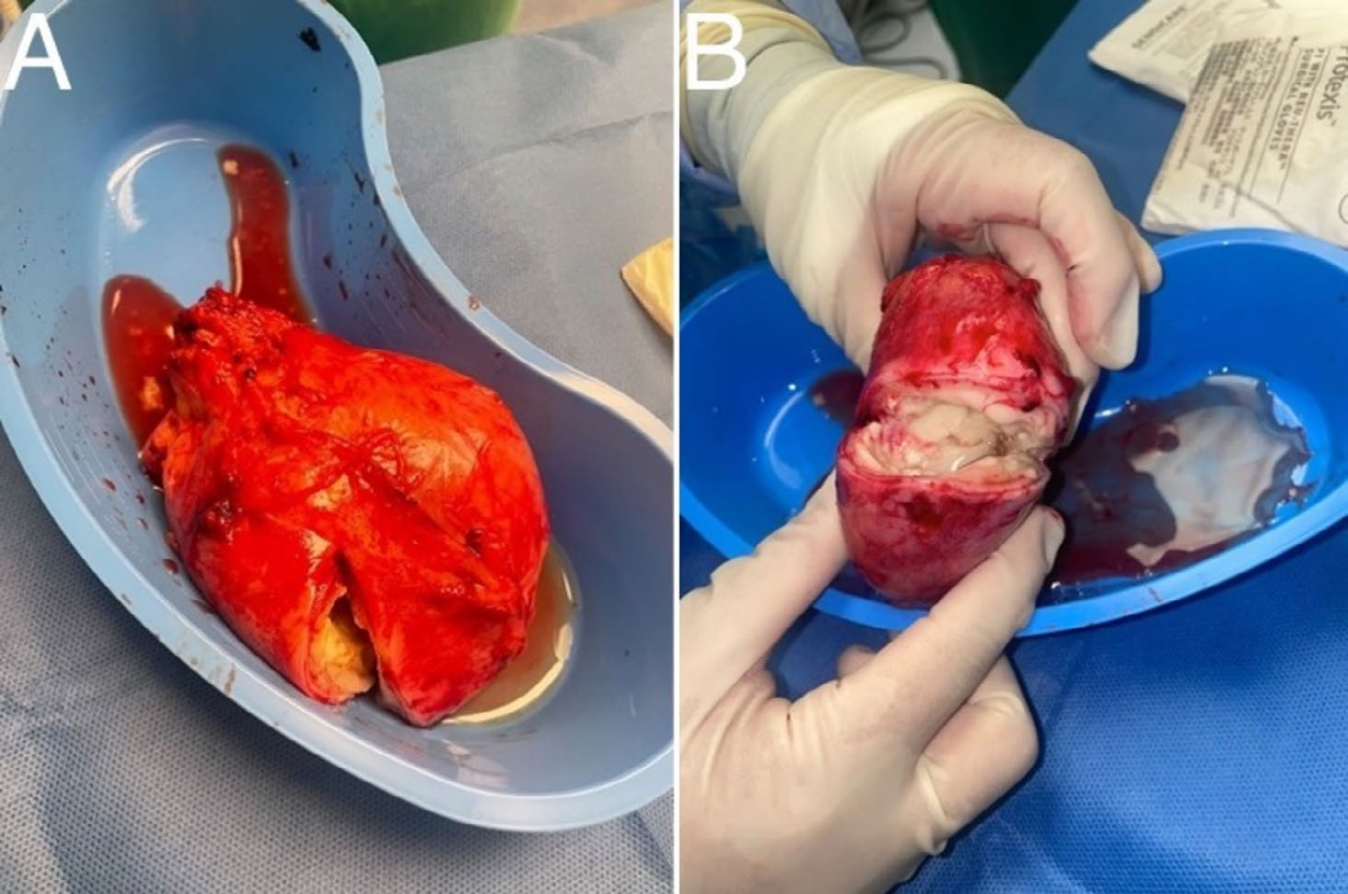
(A, B) A fibrous mass with gray tissue that was excised during right inguinal hernia repair.

**FIGURE 3:**
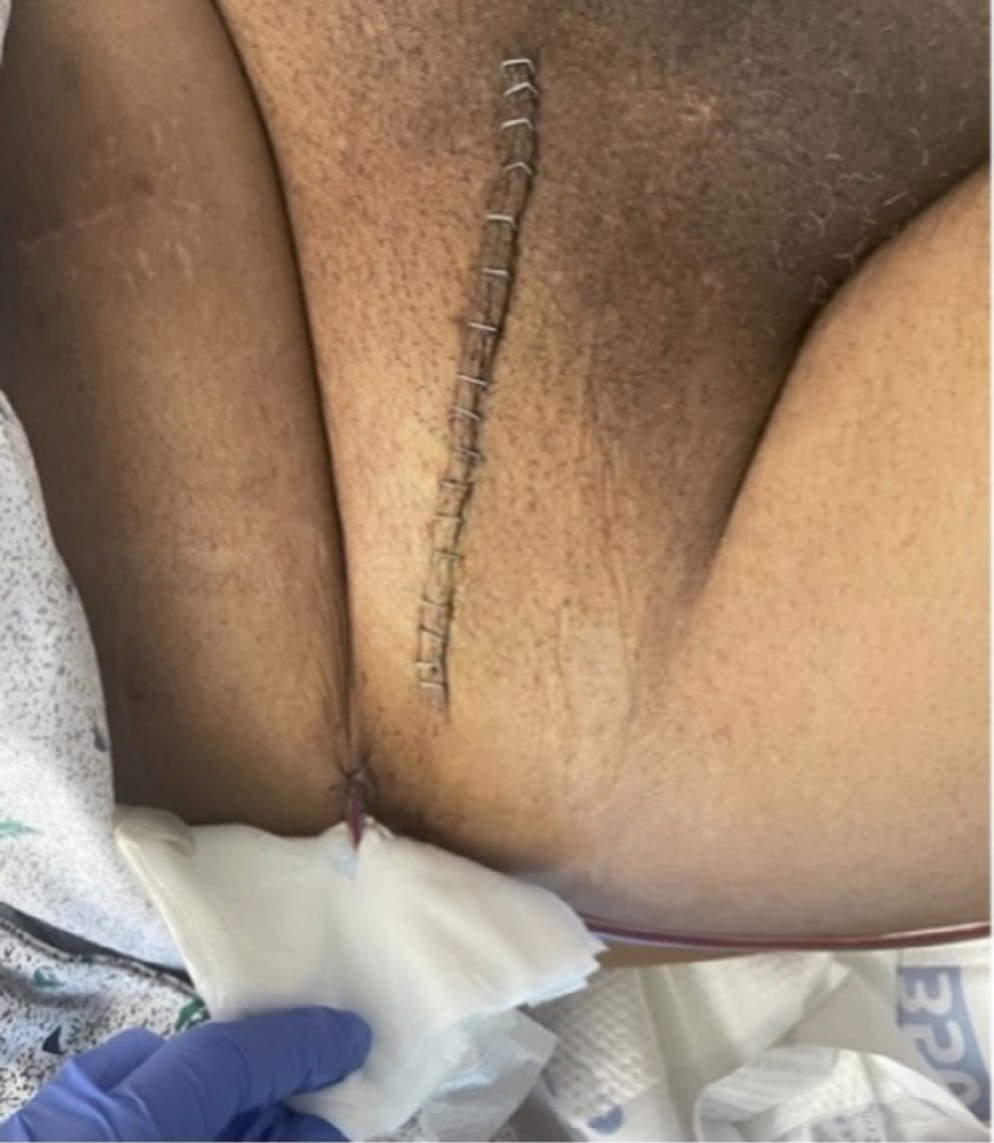
Open inguinal hernia repair closure with staples and adjacent wound drain.

On hospital Day 4, the patient reported pain from the right inguinal hernia, prompting surgical evaluation. During open hernia repair, the right testicle and a chronically inflamed mass were identified within the inguinal sac. After visualizing the incarceration by reflecting the external oblique fascia, the right testicle and a chronically inflamed structure were lifted out of the scrotum. The encapsulated structure was exposed and freed. The mass was excised and visually examined, revealing gray debris with fluid leakage, as seen in Figure 2A,B. Specimens were prepared for culture, and the mass was sent to pathology for further investigation. The testicle was inspected and deemed viable, with no injury to the vascular cord. The inguinal canal was closed with a modified Bassini technique without mesh due to concerns about the mass. A 10 Fr wound drain was inserted and secured with appropriate wound closure (Figure 3). The pathologic report of the specimen indicated the presence of metastatic mucinous carcinoma in fibrous tissue with neoplastic cells positive for pan‐cytokeratin (AE1/AE3). Pathological findings and visualization during surgery suggested metastasis of the previous appendiceal cancer to the inguinal canal.

## 4 Conclusions and Results (Outcome and Follow‐Up)

**FIGURE 4:**
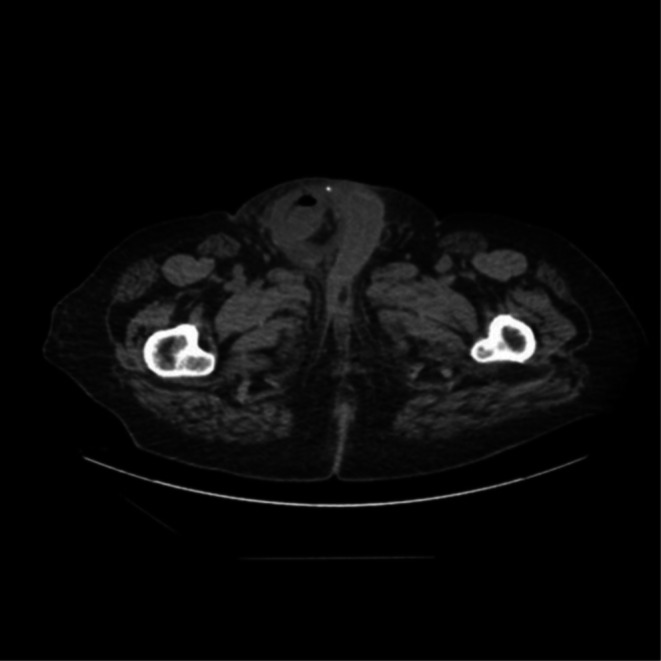
Computed tomography of the abdomen and pelvis without contrast showing fluid in the right inguinal canal with small amounts of air, with reduced size in relation to previous computed tomography in Figure 1.

Postoperatively, the patient had a decreasing fluid collection on repeat CT (Figure 4). He was discharged to rehabilitation on hospital Day 17 for continued therapy. Follow‐up with the surgical team showed adequate healing of the incision site and unremarkable findings on surveillance CT over the next 12 months.

## 5 Discussion

This case illustrates an uncommon presentation of metastatic appendiceal cancer and offers significant insight into the management and treatment of this rare pathology. Although the patient in our case was successfully treated with HIPEC and extensive resections, the temporal relationship of the hernia and previous malignancy is not fully understood. The short time between the cessation of chemotherapy and the emergency admission makes it reasonable to assume that the hernia was present but not addressed during chemotherapy due to its distance from the peritoneal cavity. The possibility of metastatic recurrence after therapeutic management is unlikely but cannot be discounted. Regardless, although the hernia sac was located outside the peritoneum, its origination from the peritoneum could have allowed for this metastatic process. This unusual case supplements the current understanding of appendiceal cancer because it highlights the possibility of metastases in patients presenting with an inguinal hernia.

Previously published cases have reported metastasis to the inguinal canal [17, 18]. In some instances, the hernia constituted the initial presentation, indicating that symptoms of a hernia could be a clinical manifestation of metastatic cancer warranting the need for surgical intervention [17]. Shimoyama et al. reported a patient who presented with an irreducible inguinal hernia as an initial sign of pseudomyxoma peritonei [18]. However, as discussed by Fitzgibbons, Jr. et al., many patients have asymptomatic hernias for which management by watchful waiting can postpone surgical repair [19]. Therefore, an asymptomatic hernia could result in metastasis being overlooked in a patient with a prior abdominal malignancy until found incidentally when surgery becomes necessary for an incarcerated hernia [14, 20]. With this knowledge, an inguinal hernia in patients with previous abdominal malignancy should be considered a clue for metastasis, and patients could potentially benefit from prompt hernia repair. Without surgical intervention in asymptomatic patients, further recognition and oncologic treatment could be delayed, allowing for further progression and worsened outcomes.

Regarding pathological testing for malignancy, the College of American Pathologists currently recommends that a microscopic examination of the hernia sac should be performed for all patients with abdominal or femoral hernias, but leaves testing of inguinal hernias to the discretion of the physician [21]. One study reported that malignancy was detected in 10 of the 455 hernia sacs that underwent pathological testing between 2010 and 2021. Of those 10 patients, seven had a previous history of abdominal cancer [22]. In another study that discussed metastatic cancer in the inguinal canal, Nicholson et al. argued that routine pathological studies of hernia sacs are not justified because the rarity of metastasis to the inguinal canal means the relative cost of the study exceeds its likely benefit [23]. Although it is not recommended or warranted to complete a microscopic examination of all hernia sacs, it could be of benefit to perform such testing in all patients with a history of abdominal malignancy to exclude tumor recurrence or spread, as the prevalence is greater in these select patients [23, 24]. Future studies could determine the necessity of histopathology of inguinal hernia sacs to rule out malignancy in those with a history of abdominal cancer.

Although our case presents an interesting and unusual presentation of appendiceal cancer metastasis, widespread metastasis is well documented in the literature, with only a limited number of reports describing metastasis to the inguinal canal. Ashcroft et al. present a patient who had tender groin swelling with ultrasound findings suggestive of an irregular, hypoechoic region in the subcutaneous tissue, indicative of a groin abscess [25]. Upon further imaging and surgical intervention, appendiceal adenocarcinoma was identified through histopathology. In a recent case report, a 64‐year‐old man was discovered to have an appendiceal carcinoid tumor inside an incarcerated Amyand's hernia sac that was found incidentally during hernia surgery [14]. Although these cases present similarly with groin abnormalities, the mechanism differed in that their patients likely had an appendix within the right inguinal canal, leading to further growth and ischemic entrapment as seen in Amyand's hernia. In contrast, our patient presentation had a previous right hemicolectomy, favoring the possibility that the metastatic lesion likely occurred from peritoneal metastasis. Regardless of the mechanisms, both case presentations highlight the risk of initial misdiagnosis in patients with appendiceal cancer similarly to our case.

Although there have been significant advancements in the treatment of pseudomyxoma peritonei with cytoreductive therapy and hyperthermic intraperitoneal chemotherapy (HIPEC), patients with appendiceal cancer are often burdened by frequent metastases and recurrences. One study from the UK concluded that 26.4% of their sample population developed a recurrence of appendiceal cancer after cytoreductive therapy and HIPEC, with only 25.5% of those patients undergoing repeat surgery [26]. A more recent study discovered a recurrence rate of 34% in their patient population, with longer post‐recurrence survival in patients with intraperitoneal recurrence compared to patients with extraperitoneal recurrence [7]. These findings underscore the unrelenting course of appendiceal cancer while emphasizing the necessity for long‐term surveillance and heightened vigilance after initial treatment.

Our case report possesses both strengths and weaknesses that merit consideration in its contribution to the current literature. Our case represents an important addition to the literature addressing appendiceal cancer and its atypical locations of metastasis. We believe that presenting this unique clinical scenario can aid healthcare providers in recognizing and offering prompt care to those with a history of appendiceal cancer. However, the limited pool of literature inhibits the ability to make recommendations and guidelines regarding patients with common presentations of hernias that have a previous history of abdominal malignancy, specifically appendiceal cancer. A broader literature collection of these presentations could allow for more focused recommendations in prognostic and therapeutic approaches.

## Author Contributions

**Peter Mounas:** conceptualization, formal analysis, investigation, writing – original draft, writing – review and editing. **Tony Elias:** investigation, methodology, writing – original draft, writing – review and editing. **Amit Kharod:** conceptualization, investigation, resources, supervision, validation, writing – review and editing. **Nikolette Zamarra:** investigation, supervision, writing – review and editing.

## Ethics Statement

Patient signed consent was received for the case report and no ethical review was required.

## Consent

Written informed consent for publication of their clinical details and/or clinical images was obtained from the patient. A copy of the consent form is available for review by the Editor of this journal.

## Conflicts of Interest

The authors declare no conflicts of interest.

## Data Availability

Data sharing is not applicable to this article as no datasets were generated or analyzed during the current study.
